# Supplementary material for: Risk variants of obesity associated genes demonstrate BMI raising effect in a large cohort
Source: PLoS One. 2022 Sep 20;17(9):e0274904. doi: 10.1371/journal.pone.0274904 (PMC9488755; doi:10.1371/journal.pone.0274904)
Supplement: S1 File — (DOCX) [file pone.0274904.s001.docx]

**Source data Saqlain *et al*.**

**Risk variants of obesity associated genes demonstrate BMI raising effect in a large cohort**

Muhammad Saqlain ^1,2^, Madiha Khalid ^1^, Muhammad Fiaz ^3^, Sadia Saeed ^1^, Asad Mehmood Raja1 ^1^, Muhammad Mobeen Zafar ^1^, Tahzeeb Fatima ^4^, João Bosco Pesquero ^5^, Cristina Maglio ^4,6,7^, Hadi Valadi ^4^, Muhammad Nawaz ^4*^, Ghazala Kaukab Raja ^1*^

^1^ University Institute of Biochemistry & Biotechnology, PMAS- Arid Agriculture University Rawalpindi, 46000, Pakistan

^2^ Department of Biochemistry and Molecular Biology, University of Sialkot, Sialkot, 51040, Pakistan

^3^ Department of Pathology, Pakistan Institute of Medical Sciences (PIMS), Islamabad, 04485, Pakistan

^4^ Department of Rheumatology and Inflammation Research, Institute of Medicine, Sahlgrenska Academy, University of Gothenburg, Gothenburg, 41346, Sweden

^5^ Center for Research and Molecular Diagnostic of Genetic Diseases – Department of Biophysics, Federal University of São Paulo, São Paulo, 04039-032, Brazil

^6^ Region Västra Götaland, Sahlgrenska University Hospital, Rheumatology Clinic, Gothenburg, 41345, Sweden

^7^ Wallenberg Center for Molecular and Translational Medicine, University of Gothenburg, Gothenburg, 41345, Sweden

**Correspondence**

Muhammad Nawaz ([muhammad.nawaz@gu.se](mailto:muhammad.nawaz@gu.se))

Department of Rheumatology and Inflammation Research, Institute of Medicine, Sahlgrenska Academy, University of Gothenburg, 413 46 Gothenburg, Sweden

Ghazala Kaukab Raja ([ghazala@uaar.edu.pk](mailto:ghazala@uaar.edu.pk))

University Institute of Biochemistry & Biotechnology, PMAS- Arid Agriculture University

Rawalpindi, Pakistan

**Source data for figure 1**

| **Gene ID** | **SNPs** | **Minor Allele (Studied Population)** | **BMI Categories Based MAFs** | | | | |
| --- | --- | --- | --- | --- | --- | --- | --- |
|  |  |  | **Normal**  **(*n*=1000)** | **Lean**  **(*n*=1000)** | **Over-weight**  **(*n*=1000)** | **Obese**  **(*n*=827)** | **Over-weight+ Obese**  **(*n*=1827)** |
| *ADIPOQ* | rs2241766 | G | 0.40 | 0.40 | 0.38 | 0.43 | 0.33 |
| *CETP* | rs3764261 | A | 0.38 | 0.38 | 0.31 | 0.36 | 0.35 |
| *FTO* | rs17817449 | G | 0.49 | 0.49 | 0.59 | 0.54 | 0.50 |
|  | rs9939609 | A | 0.39 | 0.39 | 0.51 | 0.51 | 0.51 |
|  | rs1421085 | C | 0.38 | 0.38 | 0.42 | 0.53 | 0.46 |
|  | rs1558902 | A | 0,39 | 0.39 | 0.45 | 0.63 | 0.54 |
|  | rs9941349 | T | 0.46 | 0.46 | 0.39 | 0.34 | 0.36 |
|  | rs7204609 | C | 0.05 | 0.05 | 0.04 | 0.05 | 0.05 |
| *LEP* | rs7799039 | G | 0.43 | 0.40 | 0.37 | 0.25 | 0.31 |
| *LEPR* | rs1137101 | G | 0.38 | 0.37 | 0.47 | 0.51 | 0.50 |

**Source data for figure 2**

| **Gene** | **SNP ID** | **Minor Allele** | **BMI Based Body Weight Categories** | | | |
| --- | --- | --- | --- | --- | --- | --- |
|  |  |  | **Lean (*n*=1000) (OR (95%CI)** | **p-Value** | **Overweight-Obese (*n*=1827)**  **(OR (95%CI)** | **p-Value** |
| *ADIPOQ* | rs2241766 | G | 0.48 (0.29-0.82) | 2.0x10^-4^ | 2.50 (3.1-5.40) | 3.0x10^-6^ |
| *CETP* | rs3764261 | A | 2.38 (1.30-4.34) | 1.0x10^-6^ | 0.76 (0.68-0.85) | 9.0x10^-7^ |
| *FTO* | rs17817449 | G | 0.75 (0.46-1.21) | 1.0x10^-4^ | 1.03 (1.90-2.19) | 1.0x10^-6^ |
|  | rs9939609 | A | 0.22 (0.03-1.71) | 1.0x10^-4^ | 1.44 (1.29-1.60) | 1.0x10^-6^ |
|  | rs1421085 | C | 0.93 (0.33-2.59) | 6.90x10^-1^ | 1.28 (1.16-1.41) | 1.0x10^-6^ |
|  | rs1558902 | A | 1.06 (0.38-2.95) | 8.90x10^-1^ | 3.03 (5.8-8.4) | 4.0x10^-5^ |
|  | rs9941349 | T | 1.25 (0.48-3.29) | 3.0x10^-3^ | 0.68 (0.61-0.77) | 6.0x10^-5^ |
|  | rs7204609 | C | NA | - | 1.00 (0.76-1.30) | 9.90x10^-1^ |
| *LEP* | rs7799039 | G | 1.09 (0.96-1.23) | 1.90x10^-1^ | 0.69 (0.63-0.76) | 8.0x10^-6^ |
| *LEPR* | rs1137101 | G | 1.04 (0.28-3.85) | 5.80x10^-1^ | 4.42 (2.76-7.08) | 2.0x10^-6^ |

**Source data for figure 3**

| **Genes** | **SNP ID** | **Minor Allele** | **BMI (Kg/m2) as β(SE)** | | | **p-Value** |
| --- | --- | --- | --- | --- | --- | --- |
|  |  |  | **Total Population (*n*=1827)** | **Male**  **(*n*=1799)** | **Females**  **(*n*=2028)** |  |
| *ADIPOQ* | rs2241766 | G | 0.239(0.004) | 0.227(0.0264) | 0.134(0.304) | 1.06x10^-7^ |
| *CETP* | rs3764261 | A | -0.155(0.001) | -0.142(0.183) | -0.093(0.284) | 1.8x10^-12^ |
| *FTO* | rs17817449 | G | 0.249(0.002) | 0.168(0.164) | 0.123(0.277) | 7.08x10^-8^ |
|  | rs9939609 | A | 0.314(0.002) | 0.211(0.162) | 0.259(0.280) | 4.7x10^-11^ |
|  | rs1421085 | C | 0.044(0.002) | 0.050 (0.169) | 0.035(0.265) | 0.119 |
|  | rs1558902 | A | 0.26(0.002) | 0.212(0.165) | 0.146(0.275) | 5.5x10^-23^ |
|  | rs9941349 | T | -0.125(0.001) | -0.068(0.195) | -0.110(0.302) | 1.3x10^-5^ |
|  | rs7204609 | C | 0.09(0.001) | 0.090(0.464) | 0.006(0.833) | 0.02 |
| *LEP* | rs7799039 | G | -0.103(0.002) | -0.104 (0.002) | -0.100(0.002) | 5.43x10^-8^ |
| *LEPR* | rs1137101 | G | 0.285(0.002) | 0.281(0.002) | 0.294(0.0026) | 7.5x10^-20^ |

**List of primers and sequence**

| **GENE** | **SNP** | **Primer Details** | | | | **Product Size** |
| --- | --- | --- | --- | --- | --- | --- |
|  |  | **Forward outer** | **Reverse outer** | **Allele specific (1)** | **Allele Specific (2)** |  |
| *ADIPOQ* | rs 2241766 | CTCTCCATGGCTGACAGTG | GTGCCATCTCTGCCATCAC | **F(T):**GCTATTAGCTCTGCCCGGT | **R(G):**GTGGTTTCCTGGTCATGC | T=156bp G=105bp |
| *CETP* | rs3764261 | GATTACAGGTACCTACCACCAC | GACTCCGGTATTCTTAGAAGC | **F(T):**CCTGTCGGTAGGCATCTAGT | **R(G):**AGTGAATGAGATAGCAGACGAC | Outer=477bp T=212bp G=306bp |
| *FTO* | rs 17817449 | GGCAATCTTTCTGCTTCCTG | CACACAGCAGGCATTTACAAG | **F(T):**CAGCTTGGCACACAGAAACT | **F(G):**AGCTTGGCACACAGAAACG | T and G =377bp |
|  | rs 9939609 | CATGGTGGTACGCTGCTATG | CACTCCATTTCTGACTGTTACCT | **F(A)**:TCCTTGCGACTGCTGTGAATTTA | **R(T)**:AGAGACTATCCAAGTGCATCACA | A=87bp T=306bp |
|  | rs 1421085 | FO- TTGTAATGAAGTTTTAGGCCTCAGCTTC | RO- TAAATAAATGCTTCTGGACAGTGCGTAG | **F(C)**:AGTAGCAGTTCAGGTCCTAAGGCATTAC | **R(T):**ACAAATTCTCATCAGACACTTAATCAAGGA | Outer=434bp C=201 T=291 |
|  | rs 1558902 | TCTATGAGACACTACAGGCATT | CATATCAAGTTAGGGTACGTTG | **F(A):**CCTGTGGGTTTACATTTGA | **R(T):**CAGCAATAACCTACCCAAA | Outer=90bp A=59bp T=68bp |
|  | rs 9941349 | FO-ATTATTTTAGGCTGTACTCTCCCACCT | RO- TTGCATAAAACTCTGTAACTTCATCAGC | **F(C):**ATATGATGGTTAGGTTAGGTTGCAATTC | **R(T):**AAAGTTATTCCTCTGCATATATTCCACAA | Outer=385bp C=207bp T=235bp |
|  | rs 7204609 | FO-AACACAAAAGTATGGAGTCTCAGGGCTC | RO- CAAGAAGAGGTTAGCCTAGGCTAAACCG | **F(T):**GAGCTACATCTCCTACTTAGCCGAGGTATT | **R(C):**GAGGAGACTTGCCCAGAGAGTGACAG | Outer=163bp T=119bp C=100bp |
| *LEP* | rs779903 | 5`- GCTTTCTAAGCCAAGGCAAA -3` | 5`- TTCCTGCAACATCTCAGCAC -3` | RE--HhaI | | A=226bp G=179bp |
| *LEPR* | rs1137101 | 5`-TTTTCAATATAGGCCTGAAGTGTT-3` | 5`-TTGTTAAATCATTCTAGAAGCCACTC-3` | RE --Eco91I (BstEII) | | A=391bp G=204bp, 187bp |
