# Supplementary material for: Risk variants of obesity associated genes demonstrate BMI raising effect in a large cohort
Source: PLoS One. 2022 Sep 20;17(9):e0274904. doi: 10.1371/journal.pone.0274904 (PMC9488755; doi:10.1371/journal.pone.0274904)
Supplement: Attachment — Submitted filename: PONE-D-22-10727.pdf rewied.pdf [file pone.0274904.s002.pdf]

PLOS ONE

Risk variants of obesity associated genes demonstrate BMI raising effect in a large cohort

--Manuscript Draft--

**Manuscript Number:**	PONE-D-22-10727
**Article Type:**	Research Article
**Full Title:**	Risk variants of obesity associated genes demonstrate BMI raising effect in a large cohort
**Short Title:**	Risk variants of obesity associated genes
**Corresponding Author:**	Muhammad Nawaz, PhD<br>Goteborgs Universitet<br>Gothenburg, SWEDEN
**Keywords:**	single nucleotide polymorphisms; Risk alleles; obesity; body mass index
**Abstract:**	Obesity is highly polygenic disease where several genetic variants have been reportedly associated with obesity in different ethnicities of the world. The current study we identified the risk or protective association of alleles of single nucleotide polymorphisms (SNPs) of adiponectin, C1Q and collagen domain containing (ADIPOQ), cholesteryl ester transfer protein (CEPT), FTO alpha-ketoglutarate dependent dioxygenase (FTO), leptin (LEP), and leptin receptor (LEPR) genes with obesity in a large cohort stratified into BMI-based body weight categories. The study cohort of 4000 subjects of mixed ethnicities and genders was classified into four BMI-based body weight categories i.e., normal weight, lean, over-weight, and obese. In addition to risk alleles of SNPs, the BMI raising effect of the minor allele of each genetic variant was also estimated. Based on selected candidate markers, the genotyping of all study subjects was performed by PCR assays, and genotypes and allele frequencies were calculated. The minor allele frequencies (MAFs) of all SNPs for total and BMI-based body weight categories were computed and compared with MAFs of global and South Asian (SAS) populations. Genetic associations of alleles of SNPs with BMI-based body weight categories and BMI raising risk effect for each studied genetic marker were calculated. Our data showed that in obese BMI group, the genetic variants with higher MAFs were rs2241766 (G=0.43), rs17817449 (G=0.54), rs9939609 (A=0.51), rs1421085 (C=0.53), rs1558902 (A=0.63), and rs1137101 (G=0.64) respectively. All these variants also showed statistically significant (all $p \le 0.005$), strong (OR=2.50- 4.42) to mild (OR=1.03-1.44) associations with obesity and a positive BMI raising risk effect (β= 0.239 -0.31 Kg/m 2). In contrast, three variants rs3764261 (A=0.38), rs9941349 (T=0.43) and rs7799039 (G=0.40-0.43) had higher MAFs in lean-normal BMI groups. These three SNPs also appeared to be obesity protective based on significant yet protective associations with over-weight-obese BMI (OR=0.68-0.76), and a BMI lowering effect (β= -0.103 -0.155 Kg/m 2). A significant association with lean BMI group was observed only in case of rs3764261 (OR=2.38, CI=1.30-4.34). Based on strong associations and BMI raising effect, our data have identified SNPs of ADIPOQ , FTO and LEPR genes as a set of obesity-risk markers in a studied cohort.
**Order of Authors:**	Muhammad Saqlain, PhD
	Madiha Khalid, PhD
	Muhammad Fiaz, PhD
	Sadia Saeed, PhD
	Asad Mehmood Raja, PhD
	Muhammad Mobeen Zafar, PhD
	Tahzeeb Fatima, PhD
	João Bosco Pesquero, PhD
	Cristina Maglio, PhD

Powered by Editorial Manager® and ProduXion Manager® from Aries Systems Corporation

	Hadi Valadi, PhD
	Muhammad Nawaz, PhD
	Ghazala Kaukab Raja, PhD

**Additional Information:**	
**Question**	**Response**
**Financial Disclosure**<br><br>Enter a financial disclosure statement that describes the sources of funding for the work included in this submission. Review the submission guidelines for detailed requirements. View published research articles from *PLOS ONE* for specific examples.<br><br>This statement is required for submission and **will appear in the published article** if the submission is accepted. Please make sure it is accurate.<br><br>**Unfunded studies**<br>Enter: *The author(s) received no specific funding for this work.*<br><br>**Funded studies**<br>Enter a statement with the following details:<br>• Initials of the authors who received each award<br>• Grant numbers awarded to each author<br>• The full name of each funder<br>• URL of each funder website<br>• Did the sponsors or funders play any role in the study design, data collection and analysis, decision to publish, or preparation of the manuscript?<br>• **NO** - Include this sentence at the end of your statement: *The funders had no role in study design, data collection and analysis, decision to publish, or preparation of the manuscript.*<br>• **YES** - Specify the role(s) played.<br><br>* typeset	The funders had no role in study design, data collection and analysis, decision to publish, or preparation of the manuscript.
**Competing Interests**<br><br>Use the instructions below to enter a competing interest statement for this submission. On behalf of all authors, disclose any competing interests that could be perceived to bias this	The authors have declared that no competing interests exist.

work—acknowledging all financial support and any other relevant financial or non-financial competing interests.<br><br>This statement is required for submission and **will appear in the published article** if the submission is accepted. Please make sure it is accurate and that any funding sources listed in your Funding Information later in the submission form are also declared in your Financial Disclosure statement.<br><br>View published research articles from *PLOS ONE* for specific examples.<br><br>**NO authors have competing interests**<br><br>Enter: *The authors have declared that no competing interests exist.*<br><br>**Authors with competing interests**<br><br>Enter competing interest details beginning with this statement:<br><br>*I have read the journal's policy and the authors of this manuscript have the following competing interests: [insert competing interests here]*<br><br>* typeset	
**Ethics Statement**<br><br>Enter an ethics statement for this submission. This statement is required if the study involved:<br><br>• Human participants<br>• Human specimens or tissue<br>• Vertebrate animals or cephalopods<br>• Vertebrate embryos or tissues<br>• Field research<br><br>Write "N/A" if the submission does not require an ethics statement.	The ethical approval for the study was obtained from the Ethics Committee for the Use of Human Subjects, Pir Mehr Ali Shah Arid Agriculture University Rawalpindi (PMAS-AAUR) and Pakistan Institute of Medical Sciences (PIMS), Islamabad, Pakistan. The informed written consent was signed by the subjects (participants). After getting the signed consent the data was collected on a performa by interviewing the subjects (participants). The subjects who refused to take part in the study were excluded.<br><br>The approved (i) pdf document with signatures from committee, as well as (ii) the written questionnaire that was used during sampling are available can be provided to editors if required.

Powered by Editorial Manager® and ProduXion Manager® from Aries Systems Corporation

General guidance is provided below. Consult the submission guidelines for detailed instructions. **Make sure that all information entered here is included in the Methods section of the manuscript.**<br><br>**Format for specific study types**<br><br>**Human Subject Research (involving human participants and/or tissue)**<br>• Give the name of the institutional review board or ethics committee that approved the study<br>• Include the approval number and/or a statement indicating approval of this research<br>• Indicate the form of consent obtained (written/oral) or the reason that consent was not obtained (e.g. the data were analyzed anonymously)<br><br>**Animal Research (involving vertebrate animals, embryos or tissues)**<br>• Provide the name of the Institutional Animal Care and Use Committee (IACUC) or other relevant ethics board that reviewed the study protocol, and indicate whether they approved this research or granted a formal waiver of ethical approval<br>• Include an approval number if one was obtained<br>• If the study involved *non-human primates*, add *additional details* about animal welfare and steps taken to ameliorate suffering<br>• If anesthesia, euthanasia, or any kind of animal sacrifice is part of the study, include briefly which substances and/or methods were applied<br><br>**Field Research**<br><br>Include the following details if this study involves the collection of plant, animal, or other materials from a natural setting:<br>• Field permit number<br>• Name of the institution or relevant body that granted permission	
**Data Availability**<br><br>Authors are required to make all data underlying the findings described fully	Yes - all data are fully available without restriction

Powered by Editorial Manager® and ProduXion Manager® from Aries Systems Corporation

available, without restriction, and from the time of publication. PLOS allows rare exceptions to address legal and ethical concerns. See the PLOS Data Policy and FAQ for detailed information.<br><br>A Data Availability Statement describing where the data can be found is required at submission. Your answers to this question constitute the Data Availability Statement and **will be published in the article**, if accepted.<br><br>**Important:** Stating 'data available on request from the author' is not sufficient. If your data are only available upon request, select 'No' for the first question and explain your exceptional situation in the text box.<br><br>Do the authors confirm that all data underlying the findings described in their manuscript are fully available without restriction?	
**Describe where the data may be found in full sentences. If you are copying our sample text, replace any instances of XXX with the appropriate details.**<br><br>• If the data are **held or will be held in a public repository**, include URLs, accession numbers or DOIs. If this information will only be available after acceptance, indicate this by ticking the box below. For example: *All XXX files are available from the XXX database (accession number(s) XXX, XXX.*).<br>• If the data are all contained **within the manuscript and/or Supporting Information files**, enter the following: *All relevant data are within the manuscript and its Supporting Information files.*<br>• If neither of these applies but you are able to provide **details of access elsewhere**, with or without limitations, please do so. For example:<br><br>*Data cannot be shared publicly because of [XXX]. Data are available from the XXX Institutional Data Access / Ethics*	All relevant data are within the manuscript and its Supporting Information files.

Committee (contact via XXX) for researchers who meet the criteria for access to confidential data.<br><br>*The data underlying the results presented in the study are available from (include the name of the third party and contact information or URL).*<br>• This text is appropriate if the data are owned by a third party and authors do not have permission to share the data.<br><br>* typeset	
Additional data availability information:	

Powered by Editorial Manager® and ProduXion Manager® from Aries Systems Corporation

Manuscript
Click here to access/download;Manuscript;220411_Main_Saqlain et

Risk variants of obesity associated genes demonstrate BMI raising effect in a large cohort

Muhammad Saqlain [1,2], Madiha Khalid [1], Muhammad Fiaz [3], Sadia Saeed [1], Asad Mehmood Raja [1], Muhammad Mobeen Zafar [1], Tahzeeb Fatima [4], João Bosco Pesquero [5], Cristina Maglio [4,6,7], Hadi Valadi [4], Muhammad Nawaz [4*], Ghazala Kaukab Raja [1*]

[1] University Institute of Biochemistry & Biotechnology, PMAS- Arid Agriculture University Rawalpindi, 46000, Pakistan

[2] Department of Biochemistry and Molecular Biology, University of Sialkot, Sialkot, 51040, Pakistan

[3] Department of Pathology, Pakistan Institute of Medical Sciences (PIMS), Islamabad, 04485, Pakistan

[4] Department of Rheumatology and Inflammation Research, Institute of Medicine, Sahlgrenska Academy, University of Gothenburg, Gothenburg, 41346, Sweden

[5] Center for Research and Molecular Diagnostic of Genetic Diseases – Department of Biophysics, Federal University of São Paulo, São Paulo, 04039-032, Brazil

[6] Region Västra Götaland, Sahlgrenska University Hospital, Rheumatology Clinic, Gothenburg, 41345, Sweden

[7] Wallenberg Center for Molecular and Translational Medicine, University of Gothenburg, Gothenburg, 41345, Sweden

Correspondence

Muhammad Nawaz (muhammad.nawaz@gu.se)
Department of Rheumatology and Inflammation Research, Institute of Medicine, Sahlgrenska Academy, University of Gothenburg, 413 46 Gothenburg, Sweden

Ghazala Kaukab Raja (ghazala@uaar.edu.pk)
University Institute of Biochemistry & Biotechnology, PMAS- Arid Agriculture University Rawalpindi, Pakistan

ABSTRACT

Obesity is highly polygenic disease where several genetic variants have been reportedly associated with obesity in different ethnicities of the world. The current study we identified the risk or protective association of alleles of single nucleotide polymorphisms (SNPs) of adiponectin, C1Q and collagen domain containing (*ADIPOQ*), cholesteryl ester transfer protein (*CEPT*), FTO alpha-ketoglutarate dependent dioxygenase (*FTO*), leptin (*LEP*), and leptin receptor (*LEPR*) genes with obesity in a large cohort stratified into BMI-based body weight categories. The study cohort of 4000 subjects of mixed ethnicities and genders was classified into four BMI-based body weight categories i.e., normal weight, lean, over-weight, and obese. In addition to risk alleles of SNPs, the BMI raising effect of the minor allele of each genetic variant was also estimated. Based on selected candidate markers, the genotyping of all study subjects was performed by PCR assays, and genotypes and allele frequencies were calculated. The minor allele frequencies (MAFs) of all SNPs for total and BMI-based body weight categories were computed and compared with MAFs of global and South Asian (SAS) populations. Genetic associations of alleles of SNPs with BMI-based body weight categories and BMI raising risk effect for each studied genetic marker were calculated. Our data showed that in obese BMI group, the genetic variants with higher MAFs were rs2241766 (G=0.43), rs17817449 (G=0.54), rs9939609 (A=0.51), rs1421085 (C=0.53), rs1558902 (A=0.63), and rs1137101 (G=0.64) respectively. All these variants also showed statistically significant (all $p \leq 0.005$), strong (OR=2.50- 4.42) to mild (OR=1.03-1.44) associations with obesity and a positive BMI raising risk effect (β= 0.239 -0.31 Kg/m^2). In contrast, three variants rs3764261 (A=0.38), rs9941349 (T=0.43) and rs7799039 (G=0.40-0.43) had higher MAFs in lean-normal BMI groups. These three SNPs also appeared to be obesity protective based on significant yet protective associations with over-weight-obese BMI (OR=0.68-0.76), and a BMI lowering effect (β= -0.103 -0.155 Kg/m^2). A significant association with lean BMI group was observed only in case of rs3764261 (OR=2.38, CI=1.30-4.34). Based on strong associations and BMI raising effect, our data have identified SNPs of *ADIPOQ*, *FTO* and *LEPR* genes as a set of obesity-risk markers in a studied cohort.

Introduction

Obesity is a rapidly growing health risk worldwide. Overweight and obesity represent abnormally elevated ranges of weight, generally measured in terms of body mass index (BMI). Based on BMI, the body weight is categorized into lean body mass, normal weight, overweight, and obese[1]. According to the world health organization (WHO), centers for disease control and prevention (CDC), and national institute of health (NIH) criteria, a person with BMI <18.5 Kg/m^2 will be considered either lean or underweight, 18.5-24.9 Kg/m^2 as normal weight, 25-29.9Kg/m^2 overweight, and BMI ≥30 Kg/m^2 as obese[2,3]. Out of range BMI values have been found to be associated with certain health problems, especially obesity that has been declared as the epidemic of this century[4,5]. Since 1980s, the incidence of obesity has increased up to three folds in many countries with an alarming increase in the number of affected individuals[6]. According to WHO 2016 data, there are almost 2 billion overweight and 650 million obese adults globally. If similar rates continue, it is expected that the overweight population would reach 2.7 billion and obese individuals above 1 billion by 2025[7]. This obesity epidemic has also led to a global rise in many life-threatening diseases, especially coronary heart diseases (CHD), type 2 diabetes (T2D), hypertension, among others[8]. Major factors contributing to rise in obesity are lifestyle, environmental, as well as genetic predisposition [9-12].

Common phenotypes of overweight and obesity are polygenic, heritable, and multifactorial, where most of the susceptibility to obesity is related to common DNA variants[13].Several studies have reported BMI-associated candidate genes, and related genetic polymorphisms as well as their interactions with obesogenic environmental inductions in different populations[10,14,15]. According to 2014 statistics by WHO, Pakistan has been declared the 9th country with the highest prevalence of obesity in the world with almost 5.4% subjects with obesity (>30kg/m^2) and 23% with overweight (>25kg/m^2)[16,17]. The main contributing factors are less physical activity and unhealthy eating habits, which lead to increased prevalence of obesity in Pakistan[18]. In this perspective, it is important to explore a genetic panel representing the common variants influencing BMI in this region. The research in the field of genetic markers influencing BMI phenotypes in the Pakistani populationhas been very limited. As 1000 genomes data indicates (http://www.internationalgenome.org), the allele frequencies of genetic variants widely differ among different world populations and ethnicities. The ethnicity-specific frequency distribution of these variants also tends to vary in individuals of a population/ethnic background based on

their (risk associated/or protective) susceptibility towards a disease which may be due to their exposure to population-specific natural selection[19,20].

Previously, it has been reported that the genetic variants in *ADIPOQ*, *CETP*, *FTO*, *LEP*, and *LEPR* genes are strongly correlated with obesity and associated metabolic complications in different ethnicities of the world[21-29]. Based on the potential obesogenic roles of *ADIPOQ*, *CETP*, *FTO*, *LEP*, and *LEPR* SNPs, the aim of the current study was to identify risk alleles of genetic variants in a BMI stratified Pakistani cohort by calculating MAFs, their association with obesity and BMI raising risk effect. Despite being at high risk of obesity[17], the Pakistani population lacks prior studies on genetic susceptibility to weight gain and genotype distribution data of potential obesity predisposing genetic variants. The findings of the current study could be useful in setting up a gene panel to identify individuals highly prone to weight gain as well as individualized weight management plans.

Methods

The study cohort consisted of 4000 subjects stratified into four BMI-based categories; lean, normal weight, overweight, and obese[30]. Blood samples were collected from outdoor patient department (OPD) of local hospitals in Rawalpindi and Islamabad, Pakistan. The ethical approval for the study was obtained from the Ethics Committee for the Use of Human Subjects, Pir Mehr Ali Shah Arid Agriculture University Rawalpindi (PMAS-AAUR) and Pakistan Institute of Medical Sciences (PIMS), Islamabad, Pakistan. The informed written consent was signed by the subjects (participants). After getting the signed consent the data was collected on a performa by interviewing the subjects (participants). The subjects who refused to take part in the study were excluded. Based on self-reported ethnicity, the majority of the study subjects were Punjabi with a smaller distribution of Pashtuns and Kashmiris. Anthropometric and biochemical examinations were performed in the hospital (PIMS) laboratory. This incl[illegible]s the recorded clinical assay data of age, sex, weight, height, waist circumference (WC), blood pressure (systolic blood pressure; SBP and diastolic blood pressure; DBP), fasting blood sugar (FBS[illegible] triglycerides (TG), total cholesterol (TC), high density lipoprotein cholesterol (HDL-C), low density lipoprotein cholesterol (LDL-C), and very low density lipoprotein cholesterol (vLDL-C) as described previously [8]. A ratio of body weight (in Kg) to the height (in meter square) was computed to estimate the BMI (kg/m^2). The subjects were categorized based on the WHO

defined obesity categories; lean (<18.5 Kg/m^2), normal weight (18.5-24.9 Kg/m^2), over-weight (25.0-29.9Kg/m^2), obese (≥30 Kg/m^2). The subjects who were pregnant or diagnosed with terminal health illnesses such as cancer, viral hepatitis, and advanced stage liver disorder(s) were not included in the study.

Using online databases (dbSNP, SNPedia, and 1000 genome), the SNPs of five candidate genes, *LEPR*, *LEP*, *ADIPOQ*, *FTO*, and *CETP*, were selected based on their involvement in different energy balance mechanisms and having significant association with BMI and obesity[21-26]. For genotyping, one SNP each from *LEPR*, *LEP*, *ADIPOQ*, and *CETP* genes were selected, whereas six SNPs of *FTO* gene were selected due to their widely known association with BMI and obesity.

Isolation of genomic DNA

Genomic DNA was extracted using organic solvent extraction method[31]. DNA pellet was dissolved in Tris EDTA (TE) buffer and small aliquots of DNA stocks were stored at -20 ºC. Quantification of isolated DNA was performed by NanoDrop (Cuvdrop Avan, Taiwan) microvolume spectrophotometer. The integrity of extracted DNA was confirmed on 1% agarose gel[31].

PCR based genotyping

For PCR based genotyping assays, amplification refractory mutation system-polymerase chain Reaction (Tetra-ARMS PCR) and Allele-Specific Oligonucleotide PCR (ASO-PCR) primers were designed using BATCH Primer3 and IDT SciTools[32,33]. For *LEP* and *LEPR* SNPs, restriction fragment length polymorphism (RFLP) assay was designed using web based tool NEBcutter V2.0 for the selection of restriction enzyme and expected amplified cut/un-cut SNP product size[34]. All PCR products and restriction fragments were analyzed by agarose gel electrophoresis. The list of primers used in this study are provided in supplementary material.

Statistical Analysis

Means and standard deviation (Mean±SD) were computed for all descriptive characteristics. The allele and genotype frequency distribution were calculated in overall and BMI-based subcategories. The deviation of the genotypes of all studied genetic variants from Hardy–

Weinberg equilibrium (HWE) were tested using Chi-square test (χ^2). The descriptive data was analyzed using SPSS version 21.0[35].

Logistic regression models were used to explore associations of genetic variants with BMI based obesity categories. Odds' ratios (ORs) with 95% confidence intervals (95%CI) were calculated while adjusting for age and gender to overcome the bias arising from these confounding variables. Genetic association analysis of SNPs was carried out in three BMI based groups: overweight, obese, overweight and obese combined.

The BMI raising risk effect size of the minor alleles of all genetic variants was measured by calculating β-coefficients (SE) using linear regression analysis. The β-coefficients (SE) represent the change in BMI (Kg/m^2) for each copy of the minor allele of a variant carried by an individual in total population as well as in males and females to test gender-based risk effects. Genotype/allele frequencies and association analysis for all SNPs were calculated using PLINK and SNPStats online tools[36]. To account for any error resulting from multiple SNPs tested on each sample, Bonferroni adjusted p-values ($p\text{-value} \leq 0.005$) were used for genetic association results. For all other parameters statistical significance was considered as $p\text{-value} \leq 0.05$.

Results

Characteristics of population cohort

The descriptive characteristics of population cohort are presented in **Table 1**. The clinical parameters: fasting blood sugar, triglycerides, total cholesterol, triglyceride-high density lipoprotein, triglyceride-high density lipoprotein, and triglyceride-very low-density lipoprotein were found within normal to borderline ranges (**Table 1**). The anthropometric parameters such as weight (68.0±20.30 Kg) and BMI (25.05±7.31 Kg/m^2) clearly indicated a mix of lean (28 Kg, 16 Kg/m^2), normal, overweight, and obese (145 Kg, 62.48 Kg/m^2) categories.

Table 1: Descriptive statistics of study population

Study Variables	Minimum	Maximum	Mean±SD
**Age (Years)**	20	60	35.51±10.98
**Body Weight (Kg)**	19	145	71.19±18.75
**Height (cm)**	95	210	166.14±11.89

**BMI (Kg/m^2)**	16	62[illegible]48	25.05±7.31
**WC (cm)**	25.4	220.90	98.85±30.95
**SBP (mmHg)**	81	210	133.49±19.34
**DBP (mmHg)**	60	130	82.34±10.88
**TC (mmol/L)**	1.07	61.36	4.48±1.86
**HDL (mmol/L)**	0.014	3.6	1.17±0.45
**LDL (mmol/L)**	0.09	61.07	2.97±1.85
**TG (mmol/L)**	0.3	9.77	1.73±1.02
**vLDL (mmol/L)**	0.14	1.95	0.3±0.20
**BSF (mmol/L)**	2.3	35.944	5.90±2.25

WC; waist circumference, SBP; systolic blood pressure, DBP; diastolic blood pressure, TC; Total Cholesterol, HDL; high density lipoprotein, LDL; low density lipoprotein, TG; triglycerides, vLDL; very low-density lipoprotein, FBS; fasting blood sugar

Minor allele frequencies are highly raised in total and all BMI based sub-groups of studied Pakistani cohort

The MAFs of all genetic variants in total studied population, global and SAS populations are presented in **Table 2**. The MAFs of *ADIPOQ* rs2241766 G (0.36), *CETP* A rs3764261 (0.35), *FTO* rs17817449 G (0.43), rs9939609 A (0.47), rs1421085 C (0.42), rs1558902 A (0.46), rs9941349 T (0.41), and *LEP* rs7799039 G (0.66) were higher in total studied population compared to SAS and global populations (**Table 2**). Two SNPs with MAFs lower than the global and SAS populations were, *FTO* rs7204609 C (0.04) and *LEPR* rs1137101 A (0.43). The BMI categories (Lean, over-weight, obese and over-weight-obese combined vs. normal) based comparison of the MAFs is shown in **Figure 1**. Based on BMI categories-based allele frequencies, G allele of *LEP* rs7799039 and G allele of *LEPR* rs1137101 SNPs were considered as minor alleles in current population cohort. The MAFs of following SNPs were much higher in over-weight and obese BMI categories compared to the lean and normal BMI; *FTO* rs17817449 G (0.50-0.59), rs9939609 A (0.51), rs1421085 C (0.42-0.53), rs1558902 A (0.45-0.63), and *LEPR* rs1137101 A (0.47-0.51). Whereas *ADIPOQ* SNP rs2241766 had slightly higher MAF (G=0.43) in obese category compared to normal and lean (0.40). Genetic variants with higher MAFs in lean-normal BMI categories were *CETP* A rs3764261 (0.38), *FTO* rs9941349 T (0.46), and *LEP* rs7799039 (0.40-0.43) whereas *FTO* rs7204609/C frequency was within similar ranges

among all BMI groups (**Figure 1**). The genotypes of all studied SNPs were found to be within Hardy–Weinberg equilibrium (p>0.05).

Table 2: Details of genetic variants and MAFs in global, SAS and studied population

Genes	Chromosomal Location	SNPs	Major/ Minor Allele	Global MAF	SAS MAF	Studied MAF (Total)
ADIPOQ	3q27	rs2241766	T/G	0.1514	0.151	0.36
CETP	16q21	rs3764261	C/A	0.2895	0.321	0.35
FTO	16q12.2	rs17817449	T/G	0.3077	0.289	0.43
		rs9939609	T/A	0.3401	0.288	0.47
		rs1421085	T/C	0.2286	0.307	0.42
		rs1558902	T/A	0.2280	0.307	0.46
		rs9941349	C/T	0.2710	0.367	0.41
		rs7204609	T/C	0.2181	0.072	0.04
LEP	7q31.3	rs7799039	G/A	0.4016	0.514	0.66
LEPR	1p31	rs1137101	A/G	0.5843	0.503	0.43

(Data Source: 1000 Genomes Project Phase 3). MAF; Minor Allele Frequency, SAS; South Asian population

Association analysis of genetic variants based on BMI categories

The genetic association results of studied SNPs with BMI based categories of lean and overweight-obese (combined) verses normal body weight (as a reference group) are presented in **Figure 2**. Genetic variants which showed strong associations with overweight-obese BMI category were; *LEPR* rs1137101 (OR=4.42, CI=2.76-7.08, $p=2x10^{-6}$), *FTO* rs1558902 (OR=3.03, CI=5.8-8.4, $p=4x10^{-5}$), and *ADIPOQ* rs2241766 (OR=2.50, CI=3.1-5.40, $p=3x10^{-6}$) whereas three *FTO* variants; rs9939609, rs1421085, and rs17817449 showed statistically significant yet mild associations (OR = 1.03-1.44, $p=1x10^{-6}$). In contrast, only A allele of rs3764261 *CETP* SNP showed statistically significant and a positive association with lean body mass (OR=2.38, 95% CI=1.30-4.34, $p=1x10^{-6}$). Three studied genetic variants *CETP* rs3764261 (OR=0.76, CI=0.68-0.85, $p=9x10^{-7}$), *FTO* rs9941349 (OR=0.68, CI=0.61-0.77, $p=6x10^{-5}$) and *LEP* rs7799039 (OR=0.69, CI=0.63-0.76, $p=8x10^{-5}$) showed obesity protective odds. FTO gene variant rs7204609 lacked associations with any of the BMI based obesity category (**Figure 2**).

BMI raising risk

The MAFs and association analysis results of genetic variants were further replicated to examine the BMI raising risk effect of the minor alleles of studied genetic variants (**Figure 3**). The BMI raising effect of the minor alleles of all genetic variants was measured as β coefficients (SE) representing an increase in BMI (Kg/m^2) per copy of the minor allele of a variant carried by an individual. A comparison of the β coefficients for total studied population, males and females is shown in Figure 3. The results for total studied population showed that the minor A allele of rs9939609 SNP produced highest BMI raising risk effect (β=0.314Kg/m^2, SE=0.002) followed by rs17817449 G (β=0.249Kg/m^2, SE=0.002), rs1137101 G (β=0.285Kg/m^2, SE=0.002), and rs2241766 G (β=0.239Kg/m^2, SE=0.004) alleles (**Figure 3)**.

In contrast, the A allele of rs3764261 (β= -0.155Kg/m^2, SE=0.001), T allele of rs9941349 (β= -0.125Kg/m^2, SE=0.001) and G of rs7799039 (β=-0.103Kg/m^2, SE=0.002) SNPs lacked the BMI raising effect. The trends of all BMI raising risk allele effect in males and females followed similar trends to that of total population (**Figure 3**). However, the positive BMI raising effects of rs9939609 and rs1137101 SNPs seemed more pronounced in females (β=0.259, p=$4.7x10^{-11}$ and 0.294 Kg/m^2, p=$7.5x10^{-20}$) compared to males (β=0.211, 0.281 Kg/m^2). The negative BMI raising risk allele effect of rs3764261 A allele was higher in males than female (β= -0.142, vs -0.093Kg/m^2, p=$1.8x10^{-12}$) whereas rs7799039 G allele had higher negative effect in females compared to males (β= -0.294 vs -0.281 Kg/m^2, p=$5.43x10^{-8}$).

Discussion

Despite the rapid rise in obesity and associated health complications, the identification of a set of population-specific genetic risk markers for obesity (BMI raising) has not been performed in Pakistani population. In the present study, we report identification of BMI raising risk markers, risk/MAFs, independent associations with BMI based body mass categories, and BMI raising risk effect. The minor alleles of SNPs of *LEPR*, *ADIPOQ*, and *FTO* genes showed strong association with over-weight/obese BMI (excess body weight), whereas the SNP of CEPT gene was of protective effect (lower/healthy body weight) having association with lean BMI. Of these genetic markers, risk alleles of SNPs of *LEPR*, *ADIPOQ*, and *FTO* genes showed obesogenic effect by raising BMI, whereas one SNP of *FTO* and *LEP* genes produced significant protective effect by maintaining the BMI within normal ranges. This indicates that the SNPs of *LEPR*,

ADIPOQ, and *FTO* could be considered as population specific genetic risk markers of obesity exerting their effect through anthropometric traits, BMI.

Obesity is on rise globally and there is need of identification of genetic markers with best capturing maximum disease effects [40]. The polygenic nature of human obesity, strong gene-gene, and gene to environment interactions, and above all large inter-individual and ethnic variations are potential hurdles in the discovery of accurate genetic marker(s) for human obesity. Three genes that have gained much attention are; *LEP*, *LEPR*, and *FTO*. The obesogenic roles of these genes have been documented through familial, twin and population-based studies. Of several variants in *LEP*, *LEPR*, and *FTO* genes identified and tested for obesity association, only a few have been successfully replicated in different world populations. The common disease markers in these genes have been reported to increase susceptibility towards obesity either directly or in combination with other gene(s)/variant(s).

In the present study, we report associations of ten SNPs from five candidate genes with BMI in a large Pakistani cohort. The MAFs of all genetic variants showed marked variations in total studied population, BMI based lean-obese subgroups (**Figure 1)**, global and SAS populations (**Table 2**). The MAFs of all studied genetic markers were raised in obese BMI study group (*ADIPOQ* rs2241766 G (0.43), *FTO* SNPs; rs17817449 G (0.54), rs9939609 A (0.51), rs1421085 C (0.53), rs1558902 A (0.63), and *LEPR*rs1137101 (0.51). All genotyped markers showed highly significant associations ($p<0.05$) with obesity/raised BMI (**Figure 2**). Interestingly, all genetic markers with raised MAF in obese study group also showed statistically significant and positive association with over-weight-obese BMI category (**Figure 2**). Of these markers, highly significant associations with over-weight-obese BMI category were observed in case of rs1137101, rs1558902, and rs2241766 SNPs of *LEPR*, *FTO*, and *ADIPOQ* genes (OR=2.50-4.42). Whereas three *FTO* genes SNPs rs9939609, rs1421085 and rs17817449 produced statistically significant yet mild association (OR=1.03-1.44) towards raised BMI. The risk allele effect analysis of all studied variants also confirmed positive BMI raising correlation of the minor alleles of rs1137101, rs1558902 and rs2241766 SNPs (**Figure 3**). The only exception is rs7204609, rs3764261 and rs7799039 SNPs of *FTO*, *CRPT* and *LEP* genes, where their negative β values (β= -0.155, -0.125, -0.103Kg/m^2) indicate a protective, BMI lowering effect.

The BMI raising effect size of the minor G allele of rs1137101 variant was second highest in magnitude in total studied population (β=0.285 Kg/m^2)but was highest among all studied variants when compared between male and female subjects (β=0.281-0.294 Kg/m^2, $p=7.5x10^{-20}$).Thus, our results confirm that the G allele of rs1137101*LEPR* gene SNP stands out to be an informative risk variant, increasing the susceptibility of general Pakistani populations' minor allele carriers towards weight gain. *LEPR* receptor in interactions with *LEP* gene has been reported for strong obesogenic influences in some world ethnicities while others are contradictory[37-41]. However, *LEP* gene SNP rs7799039 showed protective association with over-weight-obese BMI and a statistically significant BMI lowering effect of G allele (β =-0.103 Kg/m^2) in studied Pakistani cohort.

LEP gene has been mostly reported for its association with lipid profile especially elevated HDL levels [73]. However, the association of SNP rs3764261 of *CETP* gene with BMI is not well studied. Based on its strong association with lean BMI as well as strong BMI lowering effect in our studied population clearly indicates its possible role in maintaining healthy body weight. The estimated effect sizes of BMI raising risk alleles of all genotyped SNPs are reported in (**Figure 3**).

The rs2241766 SNP of *ADIPOQ* gene showed statistically significant association with BMI. For the BMI increasing effect size estimation, the minor G allele of rs2241766 SNP maintained high effect value (β=0.239Kg/m^2).The *ADIPOQ* gene encodes hormone adiponectin synthesized by adipocytes which helps in the metabolism of fats and carbohydrates whereby its low levels have been reported significantly correlated with elevated BMI[42-44]. The rs2241766 SNP of *ADIPOQ* is thought to be a candidate marker for obesity due to its significant association with BMI-defined obesity in various populations [45-48]. However, some contrasting reports have been linked with regional differences in frequency of *ADIPOQ* polymorphism [42].

The *FTO* gene has been shown to be strongly associated with obesity against the increase in the food intake and BMI[14,48]. Genome-wide association studies have identified associations of SNPs in the first intron of *FTO* with BMI and obesity that have been replicated in different populations and age groups [49-51]. Among *FTO* SNPs, the rs17817449 and rs9939609 are extensively studied markers. Strong association of rs17817449 with obesity/raised BMI has been reported in European, Korean, Egyptian and North Indian cohorts, whereas African Americans lacked its

association with obesity [52-57]. The rs9939609 SNP is among widely screened genetic variants in different population cohorts worldwide and is considered as a candidate marker for obesity screening [58-61]. This SNP has attracted particular interest of obesity researchers because of its strongest BMI increasing effect[62].

The rs1421085 and rs1558902 SNPs have also been reported to be strongly associated with anthropometric traits especially BMI, while excessive eating behaviors have been reported for association with rs1558902 SNP[63-65]. With regards to BMI raising effect size estimation of the risk alleles of *FTO* gene SNPs, and rs9939609 showed highest effect (β=0.314 Kg/m^2) followed by rs17817449 (β=0.249 Kg/m^2, p=1x10^{-5}) and rs1558902 (β=0.26 Kg/m^2, p=1x10^{-6}). Interestingly, thers9941349 *FTO* SNP had a negative effect (β= -0.125 Kg/m^2, p=1x10^{-4}), which clearly indicates its significant protective effect from excess weight gain/obesity perhaps via maintenance of healthy lifestyle. Our findings showing a BMI lowering, protective effect of SNP rs9941349 of *FTO* gene are contrasting with previous studies conducted on African derived population which showed correlation of polymorphism with extreme obesity[66,67].

In contrast, the rs3764261 SNP of *CETP* gene showed negative association with the BMI. The BMI effect size estimation of the risk (A) allele of this SNP showed significant decrease in BMI (β =-0.155 Kg/m^2). The *CETP* gene is mostly reported for its association with lipid profile especially elevated HDL levels[68]. However, the association of SNP rs3764261 of *CETP* gene with BMI is not well studied. Based on its strong association with lean BMI as well as strong BMI lowering effect in our studied population clearly indicates its possible role in maintaining healthy body weight. The estimated effect sizes of BMI raising risk alleles of all genotyped SNPs are reported in **Figure 3**.

Findings of present study confirm that common forms of human obesity are polygenic and could be multifactorial involving interactions among multiple genetic markers and obesogenic environmental factors [40]. Varying patterns of MAFs, genetic associations and BMI raising risk allele effect clearly indicate the complex ethnicity and demography of studied Pakistani cohort. Nevertheless, there is a need for future association studies in different Pakistani ethnic groups/subgroups considering genetic-environmental interactions along with their specific dietary and lifestyle patterns. This may help computing the individual genetic scores to assess the

risk/protective effect of a set of obesity candidate genetic markers and may aid in the development of population-based obesity screening and prevention strategies.

Availability of data and materials

All data generated or analyzed during this study are included in this published article (along with supplementary information files).

Abbreviations

ADIPOQ: Adiponectin, C1Q and collagen domain containing

BMI: Body mass index

CDC: Centers for Disease Control and Prevention

CETP: Cholesteryl ester transfer protein

CHD: Coronary heart diseases

DBP: Diastolic blood pressure

FBS: Fasting blood sugar

FTO: FTO alpha-Ketoglutarate dependent dioxygenase

HDL-C: High density lipoprotein cholesterol

LDL-C: Low density lipoprotein cholesterol

LEP: Leptin

LEPR: Leptin Receptor

MAFs: Minor allele frequencies

NIH: National Institute of Health

ORs: Odds’ ratios

RFLP: Restriction fragment length polymorphism

SAS: South Asian population

SBP: Systolic blood pressure

SNP: Single nucleotide polymorphisms

T2D: Type 2 diabetes

TC: Total Cholesterol

vLDL-C: Very low-density lipoprotein cholesterol

WHO: World Health Organization

TG: Triglycerides

Funding

This research was funded by grant 20-2255/NRPU/R&D/HEC from Higher Education Commission (HEC) Pakistan to GK Raja. The funders had no role in study design, data collection and analysis, decision to publish, or preparation of the manuscript.

Author contributions

Study concept and design: Ghazala KR and Saqlain M. Acquisition of data: Saqlain M, Madiha K, Fiaz M, Asad MR, Mobeen MZ. Analysis of data: Saqlain M, Madiha K, Mobeen MZ, Tahzeeb F, Ghazala KR, and Nawaz M. Drafting of the manuscript: Saqlain M, Madiha K, Sadia S, Ghazala KR, Nawaz M. Critical revision of the manuscript and important intellectual content: Saqlain M, Khalid M, Pesquero JB, Maglio C, Valadi H, Nawaz M, and Ghazala KR. Supervision: Ghazala KR, and Nawaz M. All authors contributed significantly to the manuscript and read and approved the final version of the manuscript.

Ethics declarations

The ethical approval for study was obtained from the Ethics Committee for the Use of Human Subjects, Pir Mehr Ali Shah Arid Agriculture University Rawalpindi (PMAS-AAUR) and Pakistan Institute of Medical Sciences (PIMS), Islamabad, Pakistan. The informed written consent was signed by the subjects (participants). After getting the signed consent the data was

collected on a performa by interviewing the subjects (participants). The subjects who refused to take part in the study were excluded.

Competing interests

The authors have declared that no competing interests exist.

Supplementary Information

All relevant data are within the manuscript and its Supporting Information files. The supplementary file contains list of primers and allele specific primer sequences. The source data for figure 1 and figure is also provided.

References

1 Ghesmaty Sangachin, M., Cavuoto, L. A. & Wang, Y. Use of various obesity measurement and classification methods in occupational safety and health research: a systematic review of the literature. *BMC Obes* **5**, 28, doi:10.1186/s40608-018-0205-5 (2018).

2 Pigeyre, M., Saqlain, M., Turcotte, M., Raja, G. K. & Meyre, D. Obesity genetics: insights from the Pakistani population. *Obes Rev* **19**, 364-380, doi:10.1111/obr.12644 (2018).

3 Herhaus, B., Kersting, A., Brahler, E. & Petrowski, K. Depression, anxiety and health status across different BMI classes: A representative study in Germany. *J Affect Disord* **276**, 45-52, doi:10.1016/j.jad.2020.07.020 (2020).

4 Gonzalez-Muniesa, P. *et al.* Obesity. *Nat Rev Dis Primers* **3**, 17034, doi:10.1038/nrdp.2017.34 (2017).

5 De Lorenzo, A. *et al.* Why primary obesity is a disease? *J Transl Med* **17**, 169, doi:10.1186/s12967-019-1919-y (2019).

6 Hruby, A. & Hu, F. B. The Epidemiology of Obesity: A Big Picture. *Pharmacoeconomics* **33**, 673-689, doi:10.1007/s40273-014-0243-x (2015).

7 Malik, V. S., Willet, W. C. & Hu, F. B. Nearly a decade on - trends, risk factors and policy implications in global obesity. *Nat Rev Endocrinol* **16**, 615-616, doi:10.1038/s41574-020-00411-y (2020).

8 Fiaz, M. *et al.* Identification of population specific risk phenotypes contributing towards development of metabolic syndrome. *Pakistan J. Zool* **48**, 949-955 (2016).

9 Brown, R. E. & Kuk, J. L. Consequences of obesity and weight loss: a devil's advocate position. *Obes Rev* **16**, 77-87, doi:10.1111/obr.12232 (2015).

10 Locke, A. E. *et al.* Genetic studies of body mass index yield new insights for obesity biology. *Nature* **518**, 197-206, doi:10.1038/nature14177 (2015).

11 Thaker, V. V. Genetic and Epigenetic Causes of Obesity. *Adolesc Med State Art Rev* **28**, 379-405 (2017).

12 Rohde, K. *et al.* Genetics and epigenetics in obesity. *Metabolism* **92**, 37-50, doi:10.1016/j.metabol.2018.10.007 (2019).

13 Lamiquiz-Moneo, I. *et al.* Genetic predictors of weight loss in overweight and obese subjects. *Sci Rep* **9**, 10770, doi:10.1038/s41598-019-47283-5 (2019).

14 Frayling, T. M. *et al.* A common variant in the FTO gene is associated with body mass index and predisposes to childhood and adult obesity. *Science* **316**, 889-894, doi:10.1126/science.1141634 (2007).

15 Willer, C. J. *et al.* Six new loci associated with body mass index highlight a neuronal influence on body weight regulation. *Nat Genet* **41**, 25-34, doi:10.1038/ng.287 (2009).

16 Asif, M., Aslam, M., Altaf, S., Atif, S. & Majid, A. Prevalence and Sociodemographic Factors of Overweight and Obesity among Pakistani Adults. *J Obes Metab Syndr* **29**, 58-66, doi:10.7570/jomes19039 (2020).

17 Ng, M. *et al.* Global, regional, and national prevalence of overweight and obesity in children and adults during 1980-2013: a systematic analysis for the Global Burden of Disease Study 2013. *Lancet* **384**, 766-781, doi:10.1016/S0140-6736(14)60460-8 (2014).

18 Samir, N., Mahmud, S. & Khuwaja, A. K. Prevalence of physical inactivity and barriers to physical activity among obese attendants at a community health-care center in Karachi, Pakistan. *BMC Res Notes* **4**, 174, doi:10.1186/1756-0500-4-174 (2011).

19 Adeyemo, A. & Rotimi, C. Genetic variants associated with complex human diseases show wide variation across multiple populations. *Public Health Genomics* **13**, 72-79, doi:10.1159/000218711 (2010).

20 Lohmueller, K. E., Mauney, M. M., Reich, D. & Braverman, J. M. Variants associated with common disease are not unusually differentiated in frequency across populations. *Am J Hum Genet* **78**, 130-136, doi:10.1086/499287 (2006).

21 Kaur, H., Badaruddoza, B., Bains, V. & Kaur, A. Genetic association of ADIPOQ gene variants (-3971A>G and +276G>T) with obesity and metabolic syndrome in North Indian Punjabi population. *PLoS One* **13**, e0204502, doi:10.1371/journal.pone.0204502 (2018).

22 Smart, M. C. *et al.* APOE, CETP and LPL genes show strong association with lipid levels in Greek children. *Nutr Metab Cardiovasc Dis* **20**, 26-33, doi:10.1016/j.numecd.2009.02.005 (2010).

23 Lan, N. *et al.* FTO - A Common Genetic Basis for Obesity and Cancer. *Front Genet* **11**, 559138, doi:10.3389/fgene.2020.559138 (2020).

24 Abdelmajed, S. S., Youssef, M., Zaki, M. E., Abu-Mandil Hassan, N. & Ismail, S. Association analysis of FTO gene polymorphisms and obesity risk among Egyptian children and adolescents. *Genes Dis* **4**, 170-175, doi:10.1016/j.gendis.2017.06.002 (2017).

25 Fujiwara, C. T., Edna de Melo, M. & Correa Mancini, M. Association of leptin gene -2548 G/A polymorphism with obesity: a meta-analysis. *Ann Nutr Metab* **66**, 109, doi:10.1159/000375253 (2015).

26 Fan, S. H. & Say, Y. H. Leptin and leptin receptor gene polymorphisms and their association with plasma leptin levels and obesity in a multi-ethnic Malaysian suburban population. *J Physiol Anthropol* **33**, 15, doi:10.1186/1880-6805-33-15 (2014).

27 Ogundele, O. E., Adekoya, K. O., Osinubi, A. A., Awofala, A. A. & Oboh, B. O. Association of adiponectin gene (ADIPOQ) polymorphisms with measures of obesity in

Nigerian young adults. *Egypt J Med Hum Genet***19**, 123-127, doi:10.1016/j.ejmhg.2017.08.005 (2018).

28 Dizaji, B. F. *et al.*Association of genetic polymorphisms of PON1 and CETP with the presence of metabolic syndrome; the effects of genotypes on their serum activity and concentrations. *Egypt J Med Hum Genet***19**, 43-48, doi:10.1016/j.ejmhg.2017.12.001 (2018).

29 Kumari, P. *et al.*Association of leptin receptor genetic variants (LEPR) with obesity and leptin level in unexplained infertility in northern Indian population. *Clin Epidemiol Glob Heal***8**, doi:10.1016/j.cegh.2019.09.003 (2020).

30 Nuttall, F. Q. Body Mass Index: Obesity, BMI, and Health: A Critical Review. *Nutr Today***50**, 117-128, doi:10.1097/NT.0000000000000092 (2015).

31 Sambrook, J. & Russell, W. D. Molecular Cloning: A Laboratory Manual. *Cold Spring Harb Lab Press Cold Spring Harb NY***1**, doi:10.1016/0092-8674(90)90210-6 (1989).

32 You, F. M. *et al.* BatchPrimer3: a high throughput web application for PCR and sequencing primer design. *BMC Bioinformatics***9**, 253, doi:10.1186/1471-2105-9-253 (2008).

33 Owczarzy, R. *et al.* IDT SciTools: a suite for analysis and design of nucleic acid oligomers. *Nucleic Acids Res***36**, W163-169, doi:10.1093/nar/gkn198 (2008).

34 Vincze, T., Posfai, J. & Roberts, R. J. NEBcutter: A program to cleave DNA with restriction enzymes. *Nucleic Acids Res***31**, 3688-3691, doi:10.1093/nar/gkg526 (2003).

35 Genomes Project, C. *et al.* A global reference for human genetic variation. *Nature***526**, 68-74, doi:10.1038/nature15393 (2015).

36 Sole, X., Guino, E., Valls, J., Iniesta, R. & Moreno, V. SNPStats: a web tool for the analysis of association studies. *Bioinformatics***22**, 1928-1929, doi:10.1093/bioinformatics/btl268 (2006).

37 Shahid, A., Rana, S., Mahmood, S. & Saeed, S. Role of leptin G-2548A polymorphism in age- and gender-specific development of obesity. *J Biosci***40**, 521-530, doi:10.1007/s12038-015-9536-2 (2015).

38 Shabana & Hasnain, S. Leptin promoter variant G2548A is associated with serum leptin and HDL-C levels in a case control observational study in association with obesity in a Pakistani cohort. *J Biosci***41**, 251-255, doi:10.1007/s12038-016-9612-2 (2016).

39 Dasgupta, S. *et al.* Genetic variants in leptin: Determinants of obesity and leptin levels in South Indian population. *Adipocyte***4**, 135-140, doi:10.4161/21623945.2014.975538 (2015).

40 Rojano-Rodriguez, M. E. *et al.* Leptin receptor gene polymorphisms and morbid obesity in Mexican patients. *Hereditas***153**, 2, doi:10.1186/s41065-016-0006-0 (2016).

41 Dougkas, A., Yaqoob, P., Givens, D. I., Reynolds, C. K. & Minihane, A. M. The impact of obesity-related SNP on appetite and energy intake. *Br J Nutr***110**, 1151-1156, doi:10.1017/S0007114513000147 (2013).

42 Lu, J. F. *et al.* Association of ADIPOQ polymorphisms with obesity risk: a meta-analysis. *Hum Immunol***75**, 1062-1068, doi:10.1016/j.humimm.2014.09.004 (2014).

43 Hu, E., Liang, P. & Spiegelman, B. M. AdipoQ is a novel adipose-specific gene dysregulated in obesity. *J Biol Chem***271**, 10697-10703, doi:10.1074/jbc.271.18.10697 (1996).

44 Phani, N. M. *et al.* Implications of critical PPARgamma2, ADIPOQ and FTO gene polymorphisms in type 2 diabetes and obesity-mediated susceptibility to type 2 diabetes

in an Indian population. *Mol Genet Genomics* **291**, 193-204, doi:10.1007/s00438-015-1097-4 (2016).

45 Apalasamy, Y. D. *et al.* Association of ADIPOQ gene with obesity and adiponectin levels in Malaysian Malays. *Mol Biol Rep* **41**, 2917-2921, doi:10.1007/s11033-014-3147-0 (2014).

46 Ramya, K., Ayyappa, K. A., Ghosh, S., Mohan, V. & Radha, V. Genetic association of ADIPOQ gene variants with type 2 diabetes, obesity and serum adiponectin levels in south Indian population. *Gene* **532**, 253-262, doi:10.1016/j.gene.2013.09.012 (2013).

47 Park, J. W., Park, J. & Jee, S. H. ADIPOQ Gene Variants Associated with Susceptibility to Obesity and Low Serum Adiponectin Levels in Healthy Koreans. *Epidemiol Health* **33**, e2011003, doi:10.4178/epih/e2011003 (2011).

48 Kalantari, N., Doaei, S., Keshavarz-Mohammadi, N., Gholamalizadeh, M. & Pazan, N. Review of studies on the fat mass and obesity-associated (FTO) gene interactions with environmental factors affecting on obesity and its impact on lifestyle interventions. *ARYA Atheroscler* **12**, 281-290 (2016).

49 Gulati, P. & Yeo, G. S. The biology of FTO: from nucleic acid demethylase to amino acid sensor. *Diabetologia* **56**, 2113-2121, doi:10.1007/s00125-013-2999-5 (2013).

50 Fonseca, A. *et al.* Genetic variants in the fat mass and obesity-associated (FTO) gene confer risk for extreme obesity and modulate adiposity in a Brazilian population. *Genet Mol Biol* **43**, e20180264, doi:10.1590/1678-4685-GMB-2018-0264 (2020).

51 Liu, C., Mou, S. & Cai, Y. FTO gene variant and risk of overweight and obesity among children and adolescents: a systematic review and meta-analysis. *PLoS One* **8**, e82133, doi:10.1371/journal.pone.0082133 (2013).

52 Barseem, N. F., El Ella, S. S. A., Tawfik, M. A. & El-Nehrawy, R. R. The Potential Implication of FTO rs17817449 Gene Polymorphism on BMI Mediated Risk For Type2 Diabetes Among Obese Egyptian Children And Adolescents. *Endocr Metab Immune Disord Drug Targets* **19**, 697-704, doi:10.2174/1871530319666190101124751 (2019).

53 Hubacek, J. A. *et al.* The FTO gene and obesity in a large Eastern European population sample: the HAPIEE study. *Obesity (Silver Spring)* **16**, 2764-2766, doi:10.1038/oby.2008.421 (2008).

54 Price, R. A., Li, W. D. & Zhao, H. FTO gene SNPs associated with extreme obesity in cases, controls and extremely discordant sister pairs. *BMC Med Genet* **9**, 4, doi:10.1186/1471-2350-9-4 (2008).

55 Cha, S. W. *et al.* Replication of genetic effects of FTO polymorphisms on BMI in a Korean population. *Obesity (Silver Spring)* **16**, 2187-2189, doi:10.1038/oby.2008.314 (2008).

56 Prakash, J. *et al.* Association of FTO rs17817449 SNP with obesity and associated physiological parameters in a north Indian population. *Ann Hum Biol* **38**, 760-763, doi:10.3109/03014460.2011.614278 (2011).

57 Wing, M. R. *et al.* Analysis of FTO gene variants with measures of obesity and glucose homeostasis in the IRAS Family Study. *Hum Genet* **125**, 615-626, doi:10.1007/s00439-009-0656-3 (2009).

58 Agagunduz, D. & Gezmen-Karadag, M. Association of FTO common variant (rs9939609) with body fat in Turkish individuals. *Lipids Health Dis* **18**, 212, doi:10.1186/s12944-019-1160-y (2019).

59 Branavan, U., Wijesundera, S., Chandrasekaran, V., Arambepola, C. & Wijeyaratne, C. In depth analysis of the association of FTO SNP (rs9939609) with the expression of classical phenotype of PCOS: a Sri Lankan study. *BMC Med Genet* **21**, 30, doi:10.1186/s12881-020-0961-1 (2020).

60 Fawwad, A., Siddiqui, I. A., Zeeshan, N. F., Shahid, S. M. & Basit, A. Association of SNP rs9939609 in FTO gene with metabolic syndrome in type 2 diabetic subjects, rectruited from a tertiary care unit of Karachi, Pakistan. *Pak J Med Sci* **31**, 140-145, doi:10.12669/pjms.311.6524 (2015).

61 Meisel, S. F., Beeken, R. J., van Jaarsveld, C. H. & Wardle, J. The Association of FTO SNP rs9939609 with Weight Gain at University. *Obes Facts* **8**, 243-251, doi:10.1159/000434733 (2015).

62 Zhao, X., Yang, Y., Sun, B. F., Zhao, Y. L. & Yang, Y. G. FTO and obesity: mechanisms of association. *Curr Diab Rep* **14**, 486, doi:10.1007/s11892-014-0486-0 (2014).

63 Albuquerque, D., Nobrega, C. & Manco, L. Association of FTO polymorphisms with obesity and obesity-related outcomes in Portuguese children. *PLoS One* **8**, e54370, doi:10.1371/journal.pone.0054370 (2013).

64 Moore, S. C. *et al.* Common genetic variants and central adiposity among Asian-Indians. *Obesity (Silver Spring)* **20**, 1902-1908, doi:10.1038/oby.2011.238 (2012).

65 Micali, N., Field, A. E., Treasure, J. L. & Evans, D. M. Are obesity risk genes associated with binge eating in adolescence? *Obesity (Silver Spring)* **23**, 1729-1736, doi:10.1002/oby.21147 (2015).

66 Hassanein, M. T. *et al.* Fine mapping of the association with obesity at the FTO locus in African-derived populations. *Hum Mol Genet* **19**, 2907-2916, doi:10.1093/hmg/ddq178 (2010).

67 Speakman, J. R., Loos, R. J. F., O'Rahilly, S., Hirschhorn, J. N. & Allison, D. B. GWAS for BMI: a treasure trove of fundamental insights into the genetic basis of obesity. *Int J Obes (Lond)* **42**, 1524-1531, doi:10.1038/s41366-018-0147-5 (2018).

68 Iwanicka, J. *et al.* Relationship between CETP gene polymorphisms with coronary artery disease in Polish population. *Mol Biol Rep* **45**, 1929-1935, doi:10.1007/s11033-018-4342-1 (2018).

Figure Legends

Figure 1: Comparison of minor allele frequencies (MAFs) based on BMI categories

The BMI categories-based distribution of the MAFs of all genetic variants is presented as bar graph. The y-axis represents frequencies of the minor alleles per BMI categories (Normal, Lean, Over-weight, Obese, and Over-weight + Obese) while genetic variants with corresponding minor alleles are shown in the x-axis. MAFs were calculated using online software SNPStats.

Figure 2: Association analysis of minor alleles with BMI stratified categories

Associations of genetic variants with BMI based categories (Lean and Over-weight + Obese) were obtained by Odds ratios (ORs) with 95% confidence intervals (95% CI). ORs (95% CI) were calculated using age and gender adjusted logistic regression models and normal BMI category considered as reference group with an OR = 1.0.

Figure 3: BMI raising risk effect size estimation of minor alleles

The BMI raising risk effect size of all genetic variants are presented as β-coefficients (SE) in total and gender based (Male vs female) population cohort obtained by linear regression analysis. The positive β-coefficient values represent an increase in BMI (Kg/m^2) while a negative value showed a decrease in BMI (Kg/m^2) for each copy of the minor allele of a variant.

Figure_1

Click here to access/download;Figure;Fig1_Comparison of MAFs based on BMI categories.jpg

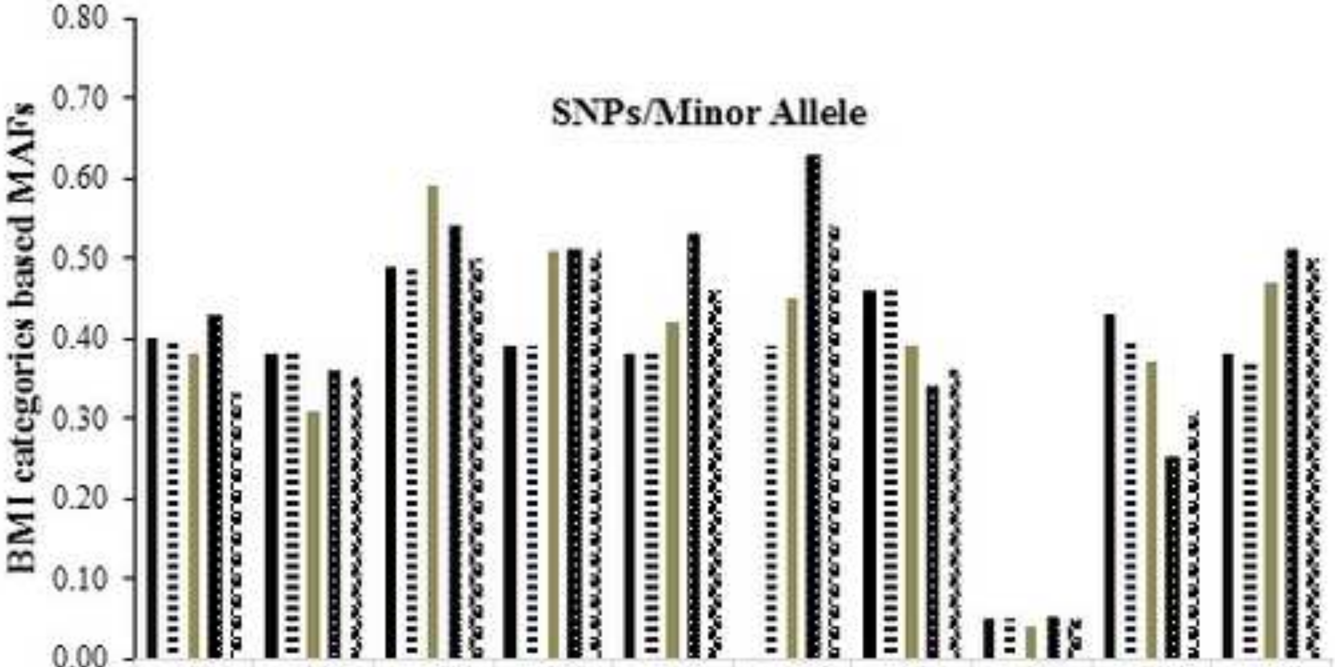

	rs2241766/G	rs3764261/A	rs17817449/G	rs9939609/A	rs1421085/C	rs1558902/A	rs9941349/T	rs7204609/C	rs7799039/G	rs1137101/G
Normal	0.40	0.38	0.49	0.39	0.38	0	0.46	0.05	0.43	0.38
Lean	0.40	0.38	0.49	0.39	0.38	0.39	0.46	0.05	0.40	0.37
Over-weight	0.38	0.31	0.59	0.51	0.42	0.45	0.39	0.04	0.37	0.47
Obese	0.43	0.36	0.54	0.51	0.53	0.63	0.34	0.05	0.25	0.51
Over-weight+ Obese	0.33	0.35	0.50	0.51	0.46	0.54	0.36	0.05	0.31	0.50

Figure_2

Click here to access/download;Figure;Fig2_Association analysis of minor alleles.jpg

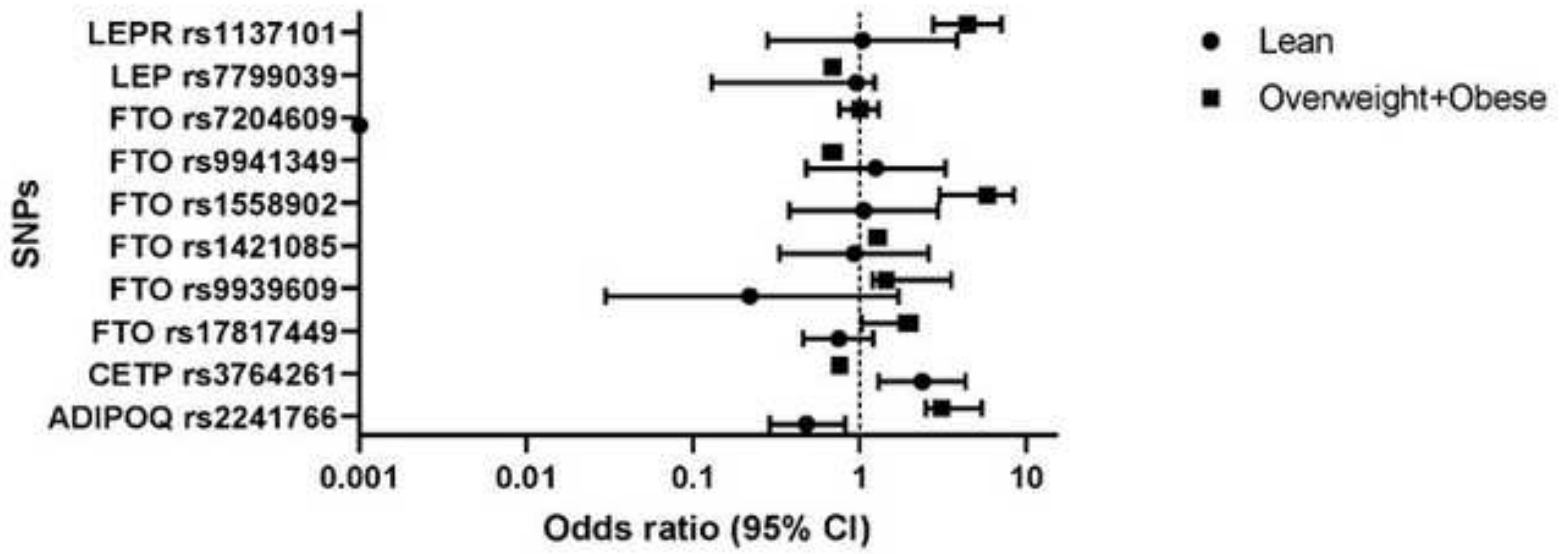

Click here to access/download;Figure;Fig3_BMI raising risk effect of minor alleles.jpg

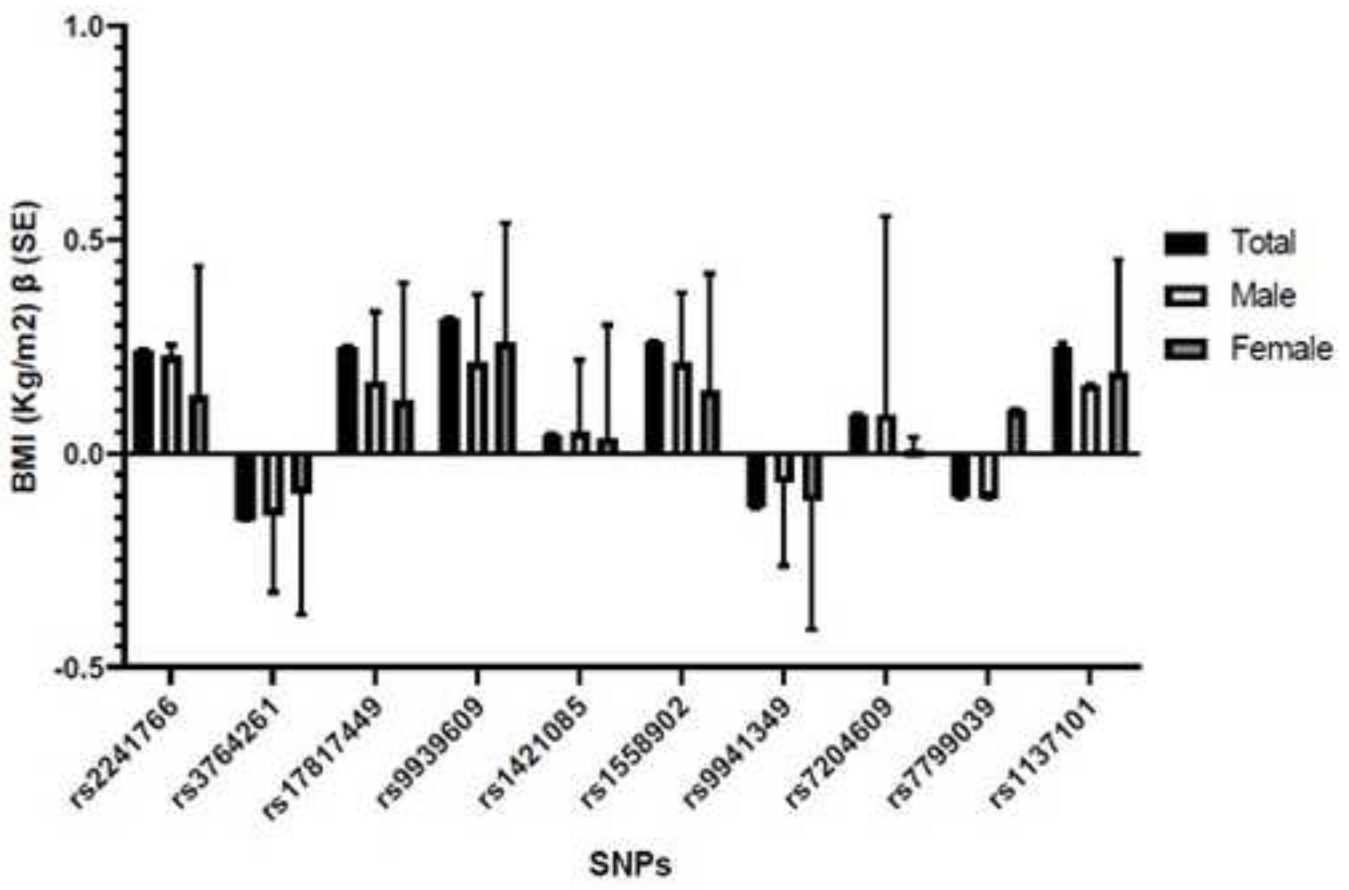

Click here to access/download
Supporting Information
220411_Source data_Saqlain et al_2022.docx
